# RNA Granules in the Mitochondria and Their Organization under Mitochondrial Stresses

**DOI:** 10.3390/ijms22179502

**Published:** 2021-09-01

**Authors:** Vanessa Joanne Xavier, Jean-Claude Martinou

**Affiliations:** Department of Cell Biology, Faculty of Sciences, University of Geneva, 1205 Geneva, Switzerland; vanessa.xavier@unige.ch

**Keywords:** mitochondrial RNA granules (MRGs), dsRNA, degradosome, nucleoids, mitochondrial gene expression, RNA processing, RNA degradation, liquid–liquid phase separation (LLPS)

## Abstract

The human mitochondrial genome (mtDNA) regulates its transcription products in specialised and distinct ways as compared to nuclear transcription. Thanks to its mtDNA mitochondria possess their own set of tRNAs, rRNAs and mRNAs that encode a subset of the protein subunits of the electron transport chain complexes. The RNA regulation within mitochondria is organised within specialised, membraneless, compartments of RNA-protein complexes, called the Mitochondrial RNA Granules (MRGs). MRGs were first identified to contain nascent mRNA, complexed with many proteins involved in RNA processing and maturation and ribosome assembly. Most recently, double-stranded RNA (dsRNA) species, a hybrid of the two complementary mRNA strands, were found to form granules in the matrix of mitochondria. These RNA granules are therefore components of the mitochondrial post-transcriptional pathway and as such play an essential role in mitochondrial gene expression. Mitochondrial dysfunctions in the form of, for example, RNA processing or RNA quality control defects, or inhibition of mitochondrial fission, can cause the loss or the aberrant accumulation of these RNA granules. These findings underline the important link between mitochondrial maintenance and the efficient expression of its genome.

## 1. Mitochondrial Gene Expression Is Compartmentalised

In almost all eukaryotic cells, mitochondria are essential organelles that are mainly known for their role in ATP production via oxidative phosphorylation (OXPHOS). OXPHOS is carried out by the electron transport chain (ETC), which consists of a series of inner membrane-embedded electron transporters forming complexes I to IV and by the ATP synthase, or complex V, which generates ATP from ADP and inorganic phosphate [1]. In mammals, the vast majority of proteins forming these complexes are encoded by nuclear genes as only 13, out of >100, are encoded by genes from the mitochondrial DNA (mt-DNA). In humans, mt-DNA is a circular molecule of 16.5 kb, encoding 37 genes. The transcripts of these genes comprise 22 tRNAs and 2 rRNAs which allow translation of the 13 mRNAs encoding the 13 proteins of the ETC [2]. The processes of the transcription and translation of mt-DNA share many characteristics and properties of the ancestral prokaryote of mitochondria. Within the mitochondrial matrix, mt-DNA is transcribed bidirectionally from both the guanine-rich Heavy and cytosine-rich Light strands (H-strand and L-strand, respectively) of the genome into long, polycistronic transcripts that are almost genome-length [3,4,5]. Most genes are encoded on the H strand whereas only MT-ND6 and 8 tRNAs are encoded by the L strand, indicating that most of the L strand is non-coding. The antisense transcripts on the L strand are often referred to as mirror RNA. The long polycistronic transcripts containing tRNAs, rRNAs and mRNAs, are processed and matured into functional transcripts. According to the so-called ‘tRNA punctuation model’, excision of tRNAs releases mRNAs and rRNAs [6]. The 5′ and 3′ of tRNAs are respectively cleaved by RNase P, which is composed of three subunits, MRPP1–3, and by RNase Z (ELAC2) [7,8]. However, the tRNA processing model is not sufficient to explain the production of tRNA-less junctions. In human mitochondria, there are several mRNAs that do not have tRNAs on both the 5′ and 3′ ends, namely, the 3′ UTR of MT-ND6; the 5′ UTR of MT-CO1; between MT-ND5 and MT-CYB mRNA; and between MT-ATP8/6, ND4-ND4L and MT-CO3 mRNAs. The catalytic enzymes and the mechanisms underlying the processing of these junctions remains poorly understood. Once released from the polycistronic transcripts, the various transcripts are matured thanks to modifications such as polyadenylation, methylation, aminoacylation or pseudouridinylation.

**Figure 1:**
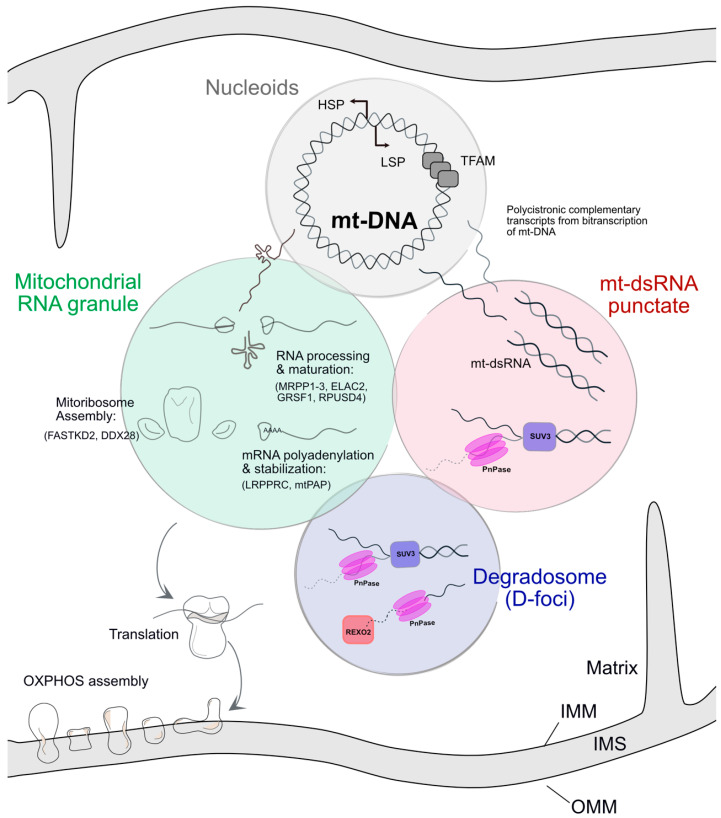
Nucleoids containing a single mt-DNA molecule, condensed by TFAM, transcribe polycistronic mRNAs. A single-stranded mRNA transcript is thought to undergo endonucleolytic processing and maturation in the Mitochondrial RNA granule (MRG). It is also the site of mitoribosome assembly. Matured mRNA transcripts transit out of the MRGs to be translated into subunits of the ETC. Bidirectional transcription of the mt-DNA from its Heavy strand promoter (HSP) and Light strand promoter (LSP) can lead to the complementary transcripts forming duplex mt-dsRNA molecules which have been observed in foci similar to MRGs. mt-dsRNA is mainly regulated by the degradosome proteins, SUV3-PNPase. The Degradosome (D-foci) is the site of mt-RNA degradation by SUV3, PNPase and REXO2. While colocalization between Nucleoids, MRGs and D-foci has been demonstrated, the association of mt-dsRNA granules to these structures has yet to be further investigated. Either they are separate entities or part of the MRGs, which would imply that the different structures are tightly connected. IMM: Inner mitochondrial membrane, IMS: Intermembrane space, OMM: Outer mitochondrial membrane.

All these complex processes do not occur in membrane limited submitochondrial compartments. Instead, mitochondria have developed specialised nucleic acid-protein rich domains that are key sites in the organization of mitochondrial gene expression (Figure 1). The most extensively studied of these membraneless structures are the nucleoids, which contain the mitochondrial genome. Each nucleoid contains one mt-DNA molecule that is densely packed with the transcription factor A, mitochondrial (TFAM) protein [9]. In mammalian cells, nucleoids average a diameter of 100 nm and are numerous within each mitochondrion [10]. In addition to TFAM, they contain a large number of proteins [11,12,13] and their integrity is required for mt-DNA’s maintenance and replication [11]. Besides nucleoids, another membraneless structure containing RNA and proteins has been identified, termed mitochondrial RNA granules (MRGs). Furthermore, very recently, new RNA structures were found, sometimes in close apposition to MRGs raising the question of whether these are subcompartments of MRGs or whether they are separate entities. The goal of this review is to describe the structure, composition and physical properties of MRGs.

## 2. Mitochondrial RNA Granules Are Formed of Large Ribonucleoprotein Complexes

In 2004 Iborra et al. [14] reported the presence of 5-BromoUridine (BrU) labelled discrete punctae along mitochondria. These punctae were first identified in T-24 urinary bladder carcinoma cells following incubation with BrU and immunostaining against BrU. These BrU positive punctae, although resembling nucleoids, were found to be lying immediately next to them within a 200 nm range. When a 60-min pulse of BrU was ‘chased’ with an excess of uridine, the intensity of the BrU-RNA foci decreased exponentially with a half-life of 45 min. This study also found that there was no correlation between the number of nucleoids and BrU-RNA foci, suggesting that some nucleoids could be transcriptionally inactive. Although this work described many key experiments about mitochondrial nascent RNA, the composition of the BrU foci was not assessed further. Ten years later, various proteins were found to colocalise with these RNA foci. A mitochondrial isoform of G-rich sequence factor 1 (GRSF1) was the first protein component to be identified to form discrete foci within the mitochondria which colocalised with BrU-labelled punctae [15,16]. It was then realized that these punctae were probably large ribonucleoprotein complexes. Because of their structure and similarity to other RNA granules in the cell, they were called mitochondria RNA granules or MRGs [15,16].

### 2.1. RNA Composition of MRGs

Until now, the precise identity of the transcripts present in the MRGs has not been revealed, and it would be interesting to know whether polycistronic, immature transcripts occupy the same or a different location as the mature transcripts ready for translation. A recent study by Zorkau et al. [17] has suggested that the mitoribosomes could be loaded with mature transcripts that are close to MRGs but not within them. The authors measured the spatiotemporal kinetics of mitochondrial protein synthesis by using fluorescent noncanonical amino acid tagging. They reported that protein synthesis was detected at sites on the cristae membranes that are distal and separate from the MRGs. However, they could not track protein synthesis with this method in real-time, and the place where mature transcripts are formed remains uncertain.

In addition, recently, using the J2 antibody, which specifically recognizes double-stranded RNA (dsRNA) [18], Dhir et al. reported the presence of dsRNA in discrete mitochondrial foci resembling MRGs [19]. The nature of the mitochondrial dsRNA (mt-dsRNA) molecules reported by Dhir et al. consisted of RNA duplexes involving all parts of the H and L strands, although their lengths were not precisely characterized.

### 2.2. Protein Composition of MRGs

As expected, a large number of proteins were found to localize within MRGs. Although previously characterized as a cytoplasmic poly(A)+ mRNA binding protein that is preferentially bound to RNA with a G-rich element [20], GRSF1 is now considered a bonafide member of the MRG proteome. Another component is the Fas-activated serine/threonine kinase (FASTK) which was identified as a protein present either in the cytosol or in MRGs, according to the translation initiation site [21]. FASTK is the prototype of a family composed of six members, FASTK and FASTKD1–5. As listed in Table 1, out of the 6 members of the FASTK family of proteins, four of them, FASTK, FASTKD1, FASTKD2 and FASTKD5 are highly enriched within the MRGs. FASTKD2 is one of the most widely used markers to label MRGs. Mutations in FASTKD2 are associated with mitochondrial encephalomyopathy, cytochrome C oxidase deficiency in skeletal muscles [22] and MELAS (Mitochondrial Encephylomyopathy, Lactic Acidosis and Stroke-like episodes)-like syndrome [23]. The silencing of FASTKD2 by RNA interference (RNAi) or by Clustered Regularly Interspaced Short Palindromic Repeats (CRISPR) caused defects mainly in the maturation, stability and assembly of the 16 S rRNA ribosome [24,25,26,27]. Downregulation of other members of the family alters the processing of specific mitochondrial RNA transcripts [28,29,30,31].

In addition to FASTK family members, several enzymes involved in mtRNA processing and maturation, as well as components of the mitoribosomes, were found to localize, at least in part, in MRGs [15,16,25,32,33,34]. Recently, using a proximity-dependent biotinylation identification (BioID) assay, Antonicka et al. generated a high-resolution network using 100 mitochondrial baits [35]. The analysis of these results showed various proteins clustering together with the main MRG protein markers, while others clustered with the proteins involved in the mitochondrial large ribosomal subunit (mtLSU) assembly or pseudouridinylation. This suggests that MRGs could be sub-compartmentalised and organized according to the various functions they are in charge of. Altogether, since the identification of GRSF1 as a marker of MRGs until today, at least 50 proteins have been proposed to localize with MRGs. It is important to note that most of these proteins are not strictly localized within MRGs as, by immunofluorescence, they can also be detected outside MRGs. This can be explained by the physical properties of MRGs as discussed later on. In Table 1, we have listed these proteins and classified them according to whether (1) they clearly form distinct punctate foci colocalizing with BrU or another bonafide MRG marker, (2) if they lie adjacent to nucleoids and are therefore presumed to be MRGs, (3) they have been described to be part of the MRG proteome by an experimental method besides microscopy. From the analysis of its proteome, it is proposed that MRGs could be the site where polycistronic transcripts accumulate, during or after transcription as they enrich many enzymes that are in charge of RNA processing and maturation. Furthermore, MRGs could be the site where mitoribosomes assemble before translation.

**Table 1:** The proteome of MRGs is tabulated below detailing their coding gene, mitochondrial function and the published experimental evidence for their localisation in MRGs.

Gene Name	Function	Experimental Evidence
ALKBH1	Histone H2A dioxygenase	Punctate foci with endogenous immunostaining [36].
C17orf80c ^1^	Uncharacterised Protein	RNA processing module of MRGs [35].
CLPX	Mitochondrial Protease subunit	
DBT	Branched-chain alpha-keto acid dehydrogenase complex subunit	
DDX28	Mitoribosome assembly	Punctate foci with overexpression of DDX28 tagged with HA [37]; endogenous immunostaining [25].
DHX30	Mitoribosome assembly	Punctate foci with endogenous immunostaining [25,38].
ELAC2 ^2^	RNAse Z: 3′ tRNA processing	Proximal punctate foci with overexpression of ELAC2 fused with Eos [39].
ERAL1	Mitoribosome assembly	Punctate foci with overexpression of ERAL1 tagged with FLAG [33].
FASTK	RNA processing	Punctate foci with overexpression of mitochondrial isoform of FASTK tagged with HA [21].
FASTKD1	RNA processing	Punctate foci with overexpression of FASTKD1 tagged with FLAG [30].
FASTKD2	RNA processing; mitoribosome assembly	Punctate foci with overexpression of FASTKD2 tagged with HA [21]. and endogenous immunostaining [25].
FASTKD5	RNA processing; mitoribosome assembly	Punctate foci with endogenous immunostaining [25]
FTSJ2 (MRM2) ^2^	rRNA methylation	Proximal punctate foci with overexpression of MRM2 fused with Eos [40].
GRSF1	RNA processing; mitoribosome assembly	Punctate foci with endogenous immunostaining [15,16]. Overexpression of mitochondrial isoform of GRSF1 [15].
GTPBP10	Mitoribosome assembly	Member of the protein interactome co-purified with DDX28 [41].
HARS2	Mitochondrial histidyl tRNA synthetase	mtLSU assembly module of MRGs [35].
HNRNPUL1 ^1^	Nuclear Ribonucleoprotein	Pseudouridylation module of MRGs [35].
HNRNPA2B1 ^1^	Nuclear Ribonucleoprotein	
HNRNPA3 ^1^	Nuclear Ribonucleoprotein	
HNRNPK ^1^	Nuclear Ribonucleoprotein	
HSD17B10 ^2^ (MRPP2)	RNAse P subunit: 5′ tRNA processing	Putative partner of GRSF1 [15]. Proximal punctate foci with overexpression of MRPP2 fused with Eos [39].
IBA57	Iron-sulfur cluster assembly factor	RNA processing module of MRGs [35].
LRPPRC	mRNA stability	Pseudouridylation module of MRGs [35].
METTL15	rRNA methylation	Punctate foci with overexpression of METTL15 tagged with FLAG [34].
MRM1 ^2^	rRNA methylation	Proximal punctate foci with overexpression of MRM1 fused with Eos [40].
MRPL12	Mitoribosome subunit	RNA processing module of MRGs [35].
MRPL14	Mitoribosome subunit	
MRPL47	Mitoribosome subunit	Punctate foci with overexpression of MRPL47 tagged with FLAG [33].
MRPL58	Mitoribosome subunit	RNA processing module of MRGs [35].
MRPS7	Mitoribosome subunit	Punctate foci with overexpression of MRPS7 tagged with FLAG [33].
MRPS9	Mitoribosome subunit	Punctate foci with overexpression of MRPS9 tagged with FLAG [33].
MTERF3 (MTERFD1)	mt-LSU assembly	Punctate foci with overexpression of MTERFD1 tagged with FLAG [34].
mtPAP	Poly(A) polymerase	Punctate foci with endogenous immunostaining and overexpression of mtPAP tagged with FLAG [32].
NFS1	Cysteine desulfurase	RNA processing module of MRGs [35].
NGRN	RNA pseudouridylation	Punctate foci with overexpression of NGRN tagged with FLAG [34].
NLRX1	Regulator of mitochondrial antiviral immunity	Punctate foci with overexpression of NLRX1 fused with GFP [42].
NOA1	Involved in mitochondrial protein translation	Punctate foci with overexpression of NOA1 tagged with FLAG [33]
NT5DC2	5′-Nucleotidase	RNA processing module of MRGs [35].
PNPT1 (PNPase)	3′-5′-exoribonuclease activity	Bimolecular fluorescence complementation of SUV3-PNPase complex partially colocalizing with MRGs [43].
PRORP (MRPP3)	RNAse P subunit: 5′ tRNA processing	Punctate foci with overexpression of MRPP3 tagged with MYC [15].
PTCD1	3′ tRNA processing	Punctate foci with overexpression of PTCD1 tagged with FLAG [33].
PTCD2	RNA processing	Punctate foci with overexpression of PTCD2 tagged with FLAG [33].
RBMX ^1^	Nuclear Ribonucleoprotein	mtLSU assembly module of MRGs [35].
RCC1L (WBSCR16)	RNA pseudouridylation	Punctate foci with overexpression of WBSCR16 tagged with FLAG [34].
RNMTL1 ^2^ (MRM3)	rRNA methylation	Proximal punctate foci with overexpression of MRM3 fused with Eos and endogenous immunostaining [40].
RPUSD3	RNA pseudouridylation	Punctate foci with overexpression of RPUSD3 tagged with FLAG [33,34].
RPUSD4	RNA pseudouridylation	Punctate foci with overexpression of RPUSD4 tagged with FLAG [33,34].
SUPV3L1 (hSUV3p)	RNA helicase	Bimolecular fluorescence complementation of SUV3-PNPase complex partially colocalizing with MRGs [43].
TFB1M	RNA modification	Punctate foci with overexpression of TFB1M tagged with FLAG [33].
TRMT10C (MRPP1)	RNAse P subunit: 5′ tRNA processing; tRNA methyltransferase	Punctate foci with overexpression of MRPPI tagged with FLAG [15].
TRUB2	RNA pseudouridylation	Punctate foci with overexpression of TRUB2 tagged with FLAG [33,34].
ZC3H4 ^1^	Zinc Finger domain protein	Pseudouridylation module of MRGs [35].
ZFR ^1^	Zinc Finger RNA Binding Protein	

^1^ These genes are not listed in the Human Mitocarta 3.0 database.^2^ Observed in foci that are adjacent to nucleoids that likely correspond to MRGs.

### 2.3. MRGs Are Sub-Compartmentalised

As mentioned above, the proteome of MRGs suggests that these structures display complex functions, which are likely to be distributed in specialized subcompartments to ensure efficacy. How MRGs are compartmentalised is a key question that can be addressed using super-resolution microscopy. Using such an approach, the diameter of MRGs, when stained for its proteins GRSF1 or FASTKD2, was found to measure ~150 nm, and when assessed using BrU staining, their size was found to be ~100 nm. The core of the MRGs would consist of compacted RNA, enveloped by RNA binding proteins (RBPs) [44]. The presence of dsRNA granules within the mitochondrial matrix further adds to the complexity of mitochondrial RNA (mt-RNA) compartmentalization, which will be detailed in Section 3. Furthermore, adjacent to MRGs, Borowski et al. [43] and Pietras et al. [45] have reported the presence of foci containing the degradosome machinery, which as its name indicates is in charge of degrading RNA. These foci were termed D-foci and will be detailed in Section 4. It is still not clear if mt-dsRNA granules are part of MRGs, D-foci or are separate entities (Figure 1). The links between these different RNA foci have yet to be fully established.

### 2.4. Physical Properties of MRGs: The Role of RNA

In mammalian cells, granules composed of RNA complexed with RBPs are found in homeostatic and stress-induced conditions. Processing bodies [46] and stress granules [47] in the cytoplasm and speckles [48] and nucleoli in the nucleus are examples of membraneless RNA granules. Recently these structures have been found to form by liquid–liquid phase separation (LLPS) and to behave like liquid condensates [49]. The exact mechanism of LLPS in biological systems leading to granule formation is not completely understood for the myriad of cellular RNA granules. Studies have shown that either the RNA [50] or proteins with intrinsically disordered domains [51] can trigger LPPS for specific types of granules (reviewed in [49]).

Rey et al. [44] have recently studied the physical properties of MRGs and demonstrated that they are fluid condensates formed by LLPS. These condensates, as expected, can fuse or fragment, and are highly dynamic as several of their components can exchange with their surroundings with high kinetics. The properties of the MRGs were found to be impacted by the dynamics of the inner mitochondrial membrane to which they are strongly associated. Similar physical properties were recently described for nucleoids [52]. If MRGs can be considered as liquid condensates, the exact mechanism of their biogenesis is yet to be understood.

**Figure 2:**
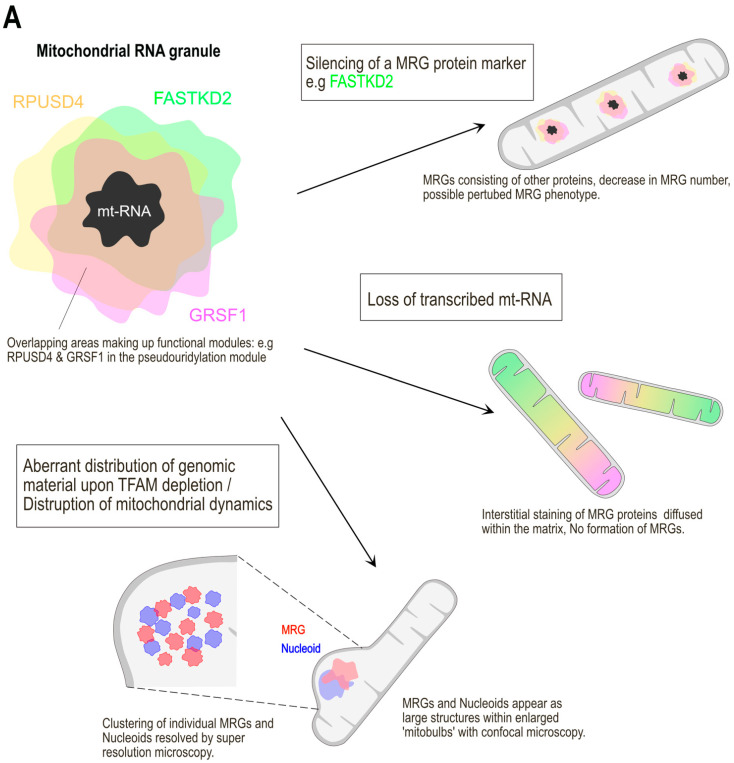

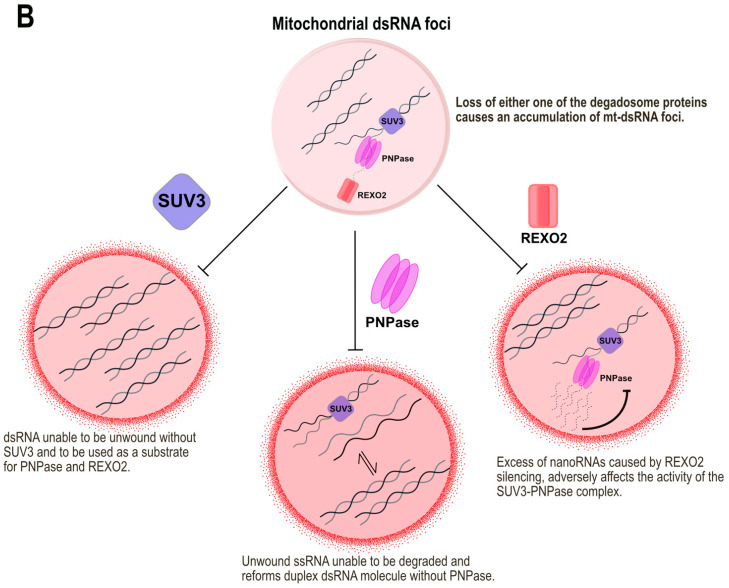
Consequences on the structure of MRGs and mt-dsRNA foci when its components are dysregulated. (**A**) Various bona fide MRG proteins phase out into liquid droplets, surrounding an RNA core in MRGs. The silencing of any one of these proteins does not destroy the persistence of MRGs in the matrix. The loss of the crucial nascent RNA core leads to the dissolution of MRGs. However, in this case, the MRG proteins can still be found diffused throughout the matrix when visualized by immunofluorescence. Enlarged mitobulbs of mitochondria are the result of disrupted membrane dynamics. Nucleoids and MRGs form tight clusters within them while maintaining their individual structures. (**B**) The loss of either one of the degradosome proteins of SUV3, PNPase and REXO2 causes an accumulation of mt-dsRNA foci in the matrix. The silencing of each of these proteins results in a different mechanism leading to the accumulation of mt-dsRNA relating to its function.

RNA has been found to be essential for the formation of MRGs (Figure 2A). Indeed, in cells devoid of mitochondrial DNA, such as in 143B ρ0, MRGs are not observed. The protein component of MRGs, GRSF1 as well as several other MRG components no longer form punctae and instead diffuse within the mitochondrial network [15,25]. Similarly, MRGs are no longer present following mt-DNA replication inhibition with Ethidium Bromide or when mt-DNA transcription is blocked with Actinomycin D [15,16] and when mt-DNA is damaged by exposure to UV irradiation [53]. In contrast, altering mitochondrial gene expression of the RNA granules downstream of transcription, using for example Chloramphenicol, which specifically blocks mitochondrial translation, does not affect MRG formation [16]. The inhibition of the activity of the ETC complex subunits I, III or V of the OXPHOS system does not affect MRGs either [25]. If RNA seems to act as a scaffold for the formation of MRGs, it remains to be determined which type of transcript, whether it is the long polycistronic or other transcripts, play this role. Furthermore, besides RNA, proteins containing disordered domains could also be involved, and their depletion would be expected to impair MRG biogenesis. Neither depletion of FASTK family members nor GRSF1 impairs the formation of MRGs. However, recently, it has been shown that the knockdown of Twinkle affects MRG formation [54]. Twinkle, the mitochondrial replication helicase that unwinds mt-DNA for replication is considered a protein component of nucleoids due to its function and its punctate colocalization with mt-DNA [55,56]. When Twinkle was used in proximity labelling experiments as a bait, several MRG associated proteins were enriched, particularly GRSF1 and MRPP1 [13,54]. These results imply that nucleoids and MRGs are tightly linked both functionally and spatially. The depletion of Twinkle with siRNA by Hensen et al. [54] led to a disruption of MRG formation both for its nascent RNA and protein component, visualized by immunostaining of BrU and GRSF1, respectively. MRG number and intensity decreased overall. However, a clear signal was observed outside of the MRGs and along the mitochondrial network for both BrU-RNA and GRSF1 which they have termed as an ‘interstitial signal’. Whether Twinkle contains low complexity domains and is able to phase separate remains to be determined.

### 2.5. Clustering of MRGs upon Impairment of Mitochondrial Dynamics

Recently, Rey et al. [44] observed that MRGs are associated with the inner mitochondrial membrane and are dependent on the dynamics of mitochondria.

Inhibition of mitochondrial fission using the overexpression of a dominant-negative mutant of the fission factor dynamic related protein 1 (Drp1_K38A) led to the formation of ‘mitobulbs’ which are enlarged domains ballooning out of an elongated network. Using super-resolved stimulated emission depletion (STED) microscopy and correlative fluorescent and electron microscopy, both nucleoids and MRGs were found to form clusters of several MRGs, similar to a bunch of grapes (Figure 2A). However, they did not fuse into a superstructure and the MRGs maintained their distinct architecture and liquid-like properties in this aberrant state. The knockdown of the mitochondrial fusion factor Mitofusin 2 (Mfn2) caused a similar clustering of MRG. The enlargement of MRGs in a pathophysiological condition had been also reported previously by Durigon et al. [57] using RNAi of the leucine zipper EF-hand-containing transmembrane protein 1 (LETM1). LETM1 is a mitochondrial inner membrane protein that is involved in the maintenance of mitochondrial tubular networks, volume and shape among its other functions [58]. Deletions in the LETM1 gene is associated with Wolf–Hirschhorn syndrome which presents a distinct facial appearance, mental retardation, growth delay, congenital hypotonia, and seizures in patients [59]. With siLETM1, MRGs—visualized by staining of both BrU and GRSF1—were enlarged in size and aberrantly distributed within the mitochondrial network. As these images were obtained by confocal microscopy, the diffraction limit of this method did not allow the authors to detect that each MRG in the cluster remained as a single entity.

In another recent study, Kleele et al. [53] studied spontaneous mitochondrial fission events and uncovered two distinct types of fission occurring either at the periphery or at the midzone of a mitochondrion. Mitochondria that fission at the periphery resulted in two asymmetric mitochondria, a smaller daughter mitochondrion breaking off from a larger daughter mitochondrion. Smaller daughter mitochondria were marked for mitophagy after fission as a method of eliminating damaged material, and as such, around 30% of them contained no MRGs post-fission. For mitochondria that underwent midzone fission, the ratio of MRGs segregated into the two mitochondrial halves were similar, presumably to be maintained within the cell. These results conclude that the overall fusion and fission dynamics of mitochondria is crucial in dictating the distribution of the MRGs across the mitochondrial network.

## 3. Granules Containing Mitochondrial Double-Stranded RNA (mt-dsRNA)

In 2018, Dhir et al. identified dsRNA molecules forming discrete foci along the mitochondrial matrix, with the use of the J2 antibody, which recognizes dsRNA of lengths > 40 nucleotides, irrespective of its sequence [19]. Importantly, the amount of dsRNA containing foci was significantly increased when the degradosome components, the DNA/RNA helicase SUV3 and the 3′-5′ exoribonuclease PNPase, were inactivated by RNAi, causing accumulations of mt-dsRNA foci in the mitochondria. The details of the mt-dsRNA accumulation by the degradosome proteins will be further discussed below in Section 5. The identification of dsRNA molecules within mitochondria is not unexpected as it had previously been reported by Aloni and Attardi using biochemical procedures [3]. They found that about 20% of the total mt-RNA fraction consisted of duplex mt-dsRNA molecules. As mentioned previously, a striking feature of mt-DNA is that both the H and L strands of its genome are transcribed fully into polycistronic transcripts. Attardi et al. had reported that the 2 strands of mt-DNA are transcribed at a comparable rate with 1–5 min pulses with radioactive uridine. With longer uridine pulses, however, the ratio of the H to L strand increased, which implies that the L strand is rapidly degraded and that less of it exists at a steady-state [3]. As the H and L transcripts are complementary, theoretically, they could form long RNA: RNA duplex molecules. However, given that the L strand is known to be rapidly and almost completely degraded by the mitochondrial degradosome, one expects most of the long polycistronic transcripts derived from the H strand, which contain most coding sequences, to be in the form of single-stranded transcripts. In addition to being processed, there is the possibility that a proportion of the H and L polycistronic transcripts hybridize and form a reservoir of long, intact, transcripts that could be processed long after transcription. Are the J2 positive discrete foci containing dsRNA molecules a nanodomain of MRGs? or a dsRNA reservoir? are questions that remain to be solved.

The binding of RNA binding proteins, during, or just after, transcription could also potentially prevent the formation of long dsRNA transcripts. Other possible RBP candidates for the regulation of mt-dsRNA are leucine-rich PPR motif-containing protein (LRPPRC), stemloop interacting RNA-binding protein (SLIRP) and mitochondrial poly(A) polymerase (MTPAP). Mutations in LRPPRC gene are responsible for the neurodegenerative disease Leigh Syndrome [60,61]. LRPPRC and SLIRP form a stable complex that has been well characterized to prevent the degradation of mtRNA and promote its stability [62]. This complex has also been shown to suppress mRNA degradation mediated by SUV3 and PNPase while promoting polyadenylation of mtRNAs by stimulating mtPAP activity [63,64]. Although LRPPRC and mtPAP have been identified as part of the MRG proteome (Table 1), the regulation of mtRNA by these proteins suggests that they may regulate mt-dsRNA as well. The study by Pajak et al. [65] is promising evidence for this. The authors investigated how defects in mt-RNA turnover lead to the accumulation of mt-dsRNA in a range of *Drosophila melanogaster* (Dm) models silenced for the Dm orthologs of SUV3, PNPase, LRPPRC and mtPAP. Their results indicated that the absence of DmPNPase, DmSUV3 and, surprisingly, DmmtPAP caused an accumulation of mt-dsRNA foci in the cytosol of dissected brains from Dm larvae. As yet, LRPPRC, SLIRP and mtPAP in mammalian models and their effects of mt-dsRNA as visualized by the J2 antibody have not been studied and are interesting research questions.

## 4. The Mitochondrial Degradosome or D-Foci

The degradosome is another specialised domain within the mitochondrial matrix, which has been suggested to be the site of mt-RNA degradation. The existence of the degradosome is highly conserved from bacteria to humans and is crucial in RNA surveillance and degradation [66]. Although first described as the RNA degrading complex in *Escherichia coli*, the degradosome machinery of mt-RNA in *Saccharomyces cerevisiae* termed mtEXO is the most well studied in eukaryotes. The mtEXO of S. cerevisiae is made up of the ATP-dependent RNA helicase, SUV3 and the exoribonuclease, DSS1 [67].

The human degradosome similarly consists of the evolutionarily conserved human orthologs: SUV3 and PNPase. SUV3 and PNPase form a complex that was visualized as foci within the mitochondrial network [43,45]. This was achieved using bimolecular fluorescence complementation (BiFC) experiments that require tagging one half of the Venus fluorescent protein to SUV3 and the other to PNPase. The interaction between the protein complex partners and the complementation of the fused Venus protein revealed discrete punctae, termed D-foci, in the matrix. The D- foci can be categorized into 4 phenotypes: (1) D-foci that colocalize with MRGs as assessed through BrU staining, (2) D-foci that colocalize with nucleoids, (3) D-foci colocalising with both MRGs and nucleoids, (4) D-foci that do not appear to associate with any other known domains. The close association of D-foci to MRGs and nucleoids implies that they share functionality and a possible flow or exchange of RNA species between them [45].

As mentioned above, transcription of the cytosine-rich L strand of the mitochondrial genome results in RNAs that are guanine-rich (G-rich). These transcripts are mostly non-coding except for MT-ND6, which is the most G-rich sequence of all the mitochondrial coding transcripts [16]. G-rich sequences are also known to form stacked structures consisting of four guanines, called G-quadruplexes (G4) [68]. Pietras et al. [45] firstly showed that GRSF1 co-purified with the degradosome proteins PNPase and SUV3. Then, these authors reported that GRSF1 foci colocalize at an occurrence of 41% with D-foci, formed by the SUV3-PNPase complex visualized thanks to the BiFC assay, as described above [43]. When the degradosome proteins were mutated to be enzymatically inactive, the occurrence of colocalization of GRSF1 foci and SUV3-PNPase D-foci increased to 78%. Pietras et al. also showed that GRSF1 can unwind G4 RNA structures, allowing the SUV3-PNPase complex to carry out efficient degradation of these non-canonical structures. Accordingly, the silencing of GRSF1 showed an accumulation of L-strand derived transcripts, albeit not as strong as the accumulation observed with the silencing of PNPase or SUV3. In conclusion, these authors proposed a model whereby GRSF1 would first melt G4 structures into linear RNA allowing better access to the degradosome for the elimination of unwanted RNA. Taken together, these results suggest strong cooperation between GRSF1, essentially the MRGs, and the degradosome. This cooperativity is also supported by the results of Hensen et al. showing that silencing of GRSF1 causes an accumulation of dsRNA foci [54], a phenotype observed when the degradosome is inhibited [19].

Most recently, REXO2, a 3′ to 5′ exonuclease, has been reported to localise to mitochondria [69], and its silencing was reported to stimulate inappropriate transcription initiation due to high levels of dinucleotides that aberrantly prime mt-DNA for transcription [70]. Therefore, these authors concluded that REXO2, through degradation of small dinucleotides, ensures normal transcription. As PNPase is unable to degrade RNA oligonucleotides that are smaller than 4 nucleotides; it is suggested that REXO2 works downstream of PNPase, breaking down small, leftover fragments. For these reasons, REXO2 can be considered a possible component of the degradosome, although it has not been shown to localise within D-foci [71,72].

The degradosome has also been shown to participate in the proper functioning of mt-DNA by preventing the accumulation of mitochondrial R loops [73]. R loops generally are structures of a DNA-RNA hybrid attached to a displaced DNA strand. Silva et al. used the S9.6 anti-DNA-RNA hybrid antibody to visualize these R loop aggregates colocalizing with the mitochondrial network in HeLa and U2OS cells. These R loop aggregates were distinct from the mt-dsRNA aggregates observed with the J2 antibody upon SUV3-PNPase silencing. The authors also showed that the R loop aggregates caused mitochondrial genomic instability by blocking mt-DNA replication. Taken together, these studies emphasize the degradosome’s importance in mt-RNA degradation and the maintenance of mt-dsRNA levels within the mitochondrial matrix, which extends to the correct functioning of overall mitochondrial gene expression starting further upstream from mt-DNA.

## 5. Dysregulation of the Degradosome and Its Effects on mt-dsRNA

As mentioned above, the dsRNA content of mitochondria is regulated by the activity of the mitochondrial degradosome, consisting of the SUV3-PNPase protein complex [19]. The loss of either one of these protein components by RNAi resulted in a marked accumulation of mt-dsRNA molecules within the mitochondria (Figure 2b). This accumulation was also observed when non-functional mutations of either protein were overexpressed, namely, the catalytically inactive dominate negative SUV3_G207V and the exonuclease activity deficient PNPase_R445E/R446E. Fibroblasts obtained from patients with pathological mutated PNPase also showed an accumulation of mt-dsRNA, as compared to fibroblasts from control patients that did not have observable mt-dsRNA. Interestingly, this increased content of dsRNA was accompanied by an interferon response that resulted from the release of dsRNA from the mitochondrial matrix into the cytosol only under the treatment of siPNPase but not siSUV3. The formation of pores via mitochondrial outer membrane permeabilization (MOMP) by BAX/BAK, proapoptotic members of the Bcl-2 family, is probably responsible for the release of mt-dsRNA into the cytosol. Similar to what has been reported for the release of mtDNA into the cytosol during late events of apoptosis [74,75] or when double-stranded brakes in mtDNA are induced [76], BAX/BAK would form a pore in the outer mitochondrial membrane, allowing herniation, then rupture, of the inner membrane. It is unlikely that the rupture of the inner mitochondrial membrane allows selective release of the matrix content into the cytosol. However, it is surprising to note that depending on the cell context, either mt-dsRNA or mtDNA is responsible for the activation of the interferon response. For example, during late events of apoptosis [74], following downregulation of TFAM and clustering of nucleoids [77], it is the mtDNA that promotes the interferon response through activation of cytosolic DNA sensors such as Cyclic GMP/AMP Synthase–Stimulator Of Interferon Genes (cGAS-STING) [78,79]. However, the presence of released mt-dsRNA and its potential to activate the immune response was not investigated in these studies. Dhir et al.’s [19] study was the first to show that in cells depleted of PNPase, it is the release of dsRNA alone that accounts for the interferon response through the Retinoic Acid Inducible Protein I- Melanoma Differentiation Associated Gene 5 (RIG-I-MDA5)-driven antiviral signalling pathway. Cells depleted of SUV3 only caused the accumulation of mt-dsRNA within mitochondria, which were retained in the matrix and did not leak out into the cytosol. The exact mechanisms underlying this specificity for PNPase silencing to stimulate an mtRNA-mediated immune response is still unclear. One reason invoked to explain this difference could be linked to the localization of PNPase in the intermembrane space of mitochondria [80]. There, PNPase could prevent dsRNA from leaving mitochondria in SUV3 deficient cells and subsequently triggering an interferon response. Another explanation, maybe more likely, is based on the observation that only silencing of PNPase is deleterious to the cell and triggers BAX/BAK-dependent mitochondrial perforation, whereas the silencing of SUV3 is not adverse enough to trigger these damage response pathways. Interestingly, mutations in PNPase have been implicated in multisystem diseases, highlighting the adverse effects of its dysfunction. These include hereditary hearing loss, encephalomyopathy, axonal and auditory neuropathy and Leigh syndrome [81,82,83]. Finally, it remains unclear why depending on the cell context, the interferon response is activated by mtRNA or mtDNA. This is unexpected and suggests that mtDNA and mtRNA molecules could be released in a specific manner. Alternatively, rapid RNA degradation, possibly by the degradosome, could favour an mtDNA-mediated immune response.

As mentioned previously, REXO2 is considered to act downstream of the SUV3-PNPase complex to degrade nanoRNA sequences that are smaller than 4 nucleotides. Szewczyk et al. [71] found that depleting REXO2 in HeLa cells with microRNA caused an accumulation in mt-dsRNA foci. They found that the expected excess of nanoRNAs that accumulate after the silencing of REXO2 adversely affects the ribonucleolytic activity of PNPase, ultimately reducing the degradosome’s ability to degrade mt-dsRNA (Figure 2b). Therefore, REXO2’s role in scavenging nanoRNAs extends to the proper functioning of mt-RNA processing. Together, the degradosome members, SUV3, PNPase and REXO2 maintain levels of mt-dsRNA thereby preventing the deleterious effects that inappropriate mt-RNA accumulation has on mitochondrial function.

## 6. Perspectives

In this review, we have highlighted the different membraneless RNA enriched granules of the mitochondria that delineate the states of processing and degradation of mt-RNA. We have also detailed how the overall mitochondrial state including the proper functioning of the nucleoids, the various mitochondrial proteins and the network dynamics have an impact on the maintenance of the RNA granules relative to their number, size and arrangement within the mitochondrial matrix. The field of mt-RNA granules is relatively young, and as of now, the majority of its study is based on in vitro experiments. Furthermore, many of the results have been obtained using cancer cell lines cell lines, and it has been reported that these structures are barely detectable in primary fibroblasts. This of course raises the question of the MRG status in non-cancer cells, such as neurons or cardiomyocytes, which are highly depend on mitochondria for their metabolism. The study of MRGs in these cells would be of key interest. Furthermore, as MRGs are sites of a high number of critical reactions involving essential enzymes, it would also be interesting to study whether pathologies associated with mutations in enzymes involved in RNA processing, degradation or in ribosome assembly lead to perturbations of the physical and structural properties of MRGs.

## Data Availability

Not applicable.

## Abbreviations

BiFC	Bimolecular fluorescence complementation
BioID	Proximity-dependent biotinylation identification
BrU	5-BromoUridine
cGAS–STING	Cyclic GMP/AMP Synthase–Stimulator of Interferon Genes
CRISPR	Clustered Regularly Interspaced Short Palindromic Repeats
Dm	Drosophila melanogaster
dsRNA	Double-stranded RNA
ETC	Electron transport chain
FASTK	Fas-activated serine/threonine kinase
G-rich	Guanine-rich
G4	G-quadruplexes
GRSF1	G-rich sequence factor 1
H-strand	Heavy strand
IMM	Inner mitochondrial membrane
IMS	Intermembrane space
L-strand	Light strand
LETM1	Leucine zipper EF-hand-containing transmembrane protein 1
LLPS	Liquid–liquid phase separation
LRPPRC	Leucine-rich PPR motif-containing protein
MELAS	Mitochondrial Encephylomyopathy, Lactic Acidosis and Stroke-like episodes
Mfn2	Mitochondrial fusion factor Mitofusin 2
MOMP	Mitochondrial outer membrane permeabilization
MRGs	Mitochondrial RNA granules
mt-DNA	Mitochondrial DNA
mt-dsRNA	Mitochondrial double-stranded RNA
mt-RNA	Mitochondrial RNA
mtLSU	Mitochondrial large ribosomal subunit
mtPAP	Poly(A) polymerase
OMM	Outer mitochondrial membrane
OXPHOS	Oxidative Phosphorylation
RBPs	RNA binding proteins
RIG-I-MDA5	Retinoic Acid Inducible Protein I- Melanoma Differentiation Associated Gene 5
RNAi	RNA interference
SLIRP	Stemloop interacting RNA-binding protein
STED	Stimulated emission depletion
TFAM	Transcription factor A, mitochondrial
